# Starch-Based Green Packaging for Cleaner Production: Degradation Control, Nanocomposite Development, and Life-Cycle Sustainability

**DOI:** 10.3390/foods15203603

**Published:** 2026-10-10

**Authors:** Marat Muratkhan, Zhadyra Muratkhan, Zhazira Mukazhanova, Bakhyt Shaimenova, Berdibek Bulashev, Saule Saduakhassova, Zhazira Zhazykbayeva, Amirsana Kiykbay, Gulnazym Ospankulova, Wei Liang

**Affiliations:** 1 Saken Seifullin Kazakh Agrotechnical University, Zhenis Avenue 62, Astana 010000, Kazakhstan; m.muratkhan@beenar.kz (M.M.); bshaymenova@mail.ru (B.S.); berdibek_aruzhan@mail.ru (B.B.); saulesaduahasova5@gmail.com (S.S.); zh.zhazykbayeva@kazatu.edu.kz (Z.Z.); sanabayq@gmail.com (A.K.); 2 Department of Agricultural Product Processing, BEENAR LABS LLP, Astana 010000, Kazakhstan; zhadyramuratkhan90@mail.ru; 3 Department of Agricultural Product Processing, Faculty of Agriculture, Manash Kozybayev North Kazakhstan University, Petropavlovsk 150000, Kazakhstan; 4 Department of Chemistry, Sarsen Amanzholov East Kazakhstan University, Ust-Kamenogorsk 070010, Kazakhstan; mukazhanovazhb@mail.ru; 5 State Key Laboratory of Marine Food Processing & Safety Control, School of Food Science and Technology, National Engineering Research Center of Seafood, Dalian Polytechnic University, Dalian 116034, China

**Keywords:** starch, cleaner production, green packaging, degradation kinetics, starch-based nanocomposites, life cycle assessment, circular economy

## Abstract

Petroleum-based packaging remains a major source of fossil-resource consumption, greenhouse-gas emissions, and persistent plastic waste, creating a need for packaging systems aligned with cleaner production and circular-economy principles. Starch is an abundant, low-cost, and biodegradable biopolymer, but its moisture sensitivity, weak mechanical performance, and poorly controlled degradation limit large-scale substitution of conventional plastics. This review synthesizes recent progress in starch-based packaging from the perspective of degradation control, nanocomposite development, and life-cycle sustainability. The multiscale structure of starch is first linked to enzymatic accessibility and environmental degradation kinetics. Biological, physical, and chemical degradation pathways are then discussed in relation to service stability and end-of-life disintegration. Strategies involving individual nanofillers, synergistic combinations of nanofillers, and environmentally friendly nanocomposite preparation are evaluated for their ability to improve barrier and mechanical performance while regulating degradation behavior. Life-cycle assessment, carbon-footprint accounting, and degradation-product safety are further integrated to identify trade-offs among material performance, processing emissions, agricultural inputs, nanofiller fate, and disposal scenarios. The review highlights that cleaner production of starch-based packaging requires simultaneous optimization of renewable feedstocks, low-carbon modification processes, controllable degradation, and standardized environmental assessment. Future development should prioritize scalable and environmentally friendly production of starch-based nanocomposites, smart degradation triggers, multi-scenario life-cycle evaluation, and industrial processing windows to support verifiable environmental benefits.

## 1. Introduction

Since the mid-20th century, petroleum-based plastics have become extensively used in the packaging industry because of their low density, cost-effectiveness, favorable mechanical properties, and versatile processability [1,2]. Commodity thermoplastics such as polyethylene (PE), polypropylene (PP), and polyethylene terephthalate (PET), together with functional polymers such as polyvinyl alcohol (PVA), are widely used in food, household-product, personal-care, and medical packaging owing to their favorable transparency, heat-sealability, and barrier properties [3]. This combination of functional performance and relatively low production cost has driven global plastic production to hundreds of millions of metric tons annually. Within this expanding market, packaging represents the largest end-use sector and accounts for a substantial proportion of global plastic consumption [4].

However, the widespread adoption of petroleum-based plastic packaging, despite the convenience it provides, has increasingly exposed the inherent limitations of the prevailing linear economic model [5]. Historical estimates indicate that only approximately 9% of all plastic waste generated has been recycled, while most of the remaining waste has been landfilled, incinerated, or released into the natural environment [6]. Polyethylene films may persist for decades or even centuries under ambient conditions, whereas the gradual fragmentation of plastic products generates microplastics that accumulate in terrestrial and aquatic ecosystems and have also been detected in human placental tissue [7,8]. Furthermore, the production and incineration of petroleum-based plastics generate substantial greenhouse-gas emissions and contribute to the continued depletion of fossil resources [9]. This linear model, characterized by resource extraction, single-use consumption, and uncontrolled disposal, is fundamentally inconsistent with global carbon-neutrality objectives and the principles of a circular economy.

In response to intensifying plastic pollution, the international community has accelerated the development of regulatory frameworks aimed at steering the packaging industry toward recyclable, reusable, and environmentally sustainable solutions [10]. In parallel, research programs have increasingly emphasized bio-based and biodegradable packaging materials, including nanomaterial-modification strategies designed to improve their functional performance [11]. Driven by these policy and technological developments, renewable biopolymers characterized by resource renewability, inherent biodegradability, and a potentially lower carbon footprint have emerged as promising alternatives to conventional petroleum-based plastics [12]. Among these materials, starch occupies a distinctive position because of its abundance, accessibility, film-forming capacity, and considerable potential for biodegradable packaging applications [13].

In the development of starch-based nanocomposites, functional nanofillers such as cellulose nanocrystals, chitosan nanofibers, layered double hydroxides, and metal oxide nanoparticles have been incorporated into starch matrices to improve their mechanical properties, water vapor barrier performance, and thermal stability [14,15,16]. Research on degradation kinetics has examined the degradation behavior of starch-based materials in soil, freshwater, and marine environments, establishing preliminary relationships among degradation rate, material composition, and environmental conditions [17]. In parallel, life cycle assessment has been applied to quantify the carbon footprint, resource consumption, and potential ecotoxicological impacts of starch-based materials throughout their life cycles, from raw-material acquisition and manufacturing to use and end-of-life treatment [18]. Collectively, these advances have supported the transition of starch-based biodegradable packaging from laboratory-scale development toward practical applications.

Despite this progress, practical and environmental challenges remain. Packaging stability during use must be balanced against predictable degradation after disposal [19]. Nanofiller dispersion and interfacial compatibility can require additional processing and increase costs [20], while environmental assessments need to account more fully for nanofiller ecotoxicity, degradation-product fate, and realistic end-of-life conditions [21]. Together, these issues limit industrial implementation.

More fundamentally, the existing literature remains fragmented. Many investigations address isolated aspects of starch-based packaging, including mechanical reinforcement without considering degradation behavior, empirical degradation rates without identifying structural and mechanistic relationships, or environmental assessments disconnected from material design and processing conditions [22,23]. This compartmentalized approach has limited the systematic understanding of how starch structure and nanofiller incorporation influence degradation kinetics, production efficiency, and life-cycle sustainability. Consequently, an integrated framework is required to connect material design and functional performance with post-use degradation and system-level environmental impacts.

Accordingly, this review examines starch-based biodegradable materials in the context of food packaging and preservation, integrating recent advances in degradation control, nanocomposite development, and life-cycle assessment. It first explains how starch structure and material formulation influence mechanical integrity, moisture and gas barrier performance, and degradation behavior. These properties are then considered in relation to the requirements of different food categories, including fresh produce, meat, seafood, dairy products, and processed foods. Particular attention is given to the conditions under which packaging contacts food, the preservation functions reported in application studies, and the extent to which measured outcomes support claims of shelf-life extension. Finally, the review considers whether improved packaging performance can be achieved alongside practical processing and verifiable environmental benefits. By connecting material design with food-specific performance and end-of-life considerations, this review aims to identify both the potential and the remaining limitations of starch-based packaging for food applications.

This narrative review draws on the literature identified through searches of Web of Science, Scopus, and Google Scholar, with the last search update conducted in September 2026. Search terms included “starch-based packaging”, “starch-based films”, “nanocomposites”, “nanofillers”, “biodegradation”, “degradation kinetics”, “food packaging”, “cleaner production”, and “life cycle assessment”, used in combinations relevant to each topic. Particular attention was given to studies published during the preceding five years, while earlier publications were retained to explain foundational concepts and established mechanisms. The literature was selected according to its relevance to starch structure, nanocomposite development, degradation behavior, food packaging applications, and environmental assessment. Reference lists of relevant articles were also examined to identify additional publications. The selected literature was synthesized thematically to connect material design and packaging performance with degradation control and life-cycle sustainability.

## 2. Structural Characteristics of Starch and Its Role in Biodegradable Packaging

### 2.1. Chemical and Physical Structure of Starch

The biodegradability of starch originates from its molecular architecture. The α-(1 → 4) and α-(1 → 6) glycosidic linkages in starch chains are susceptible to cleavage by glycoside hydrolases, enabling the enzymatic depolymerization and subsequent biodegradation of starch under biologically active soil, aquatic, and composting conditions [24,25]. However, this degradation does not occur uniformly and is strongly governed by the multiscale structural organization of starch. Molecular-chain conformation, crystalline architecture, lamellar organization, and granular morphology collectively influence water penetration, enzyme adsorption, and the accessibility of glycosidic bonds, thereby determining the kinetics of hydrolytic cleavage [26,27].

At the molecular level, starch consists primarily of amylose and amylopectin. Amylose is a predominantly linear macromolecule composed of glucose units connected by α-(1 → 4) glycosidic bonds, whereas amylopectin has a highly branched structure containing α-(1 → 6) branch points along an α-(1 → 4)-linked backbone [28]. These molecular chains subsequently assemble into supramolecular structures. The side chains of amylopectin form double helices stabilized by hydrogen bonding and are organized into alternating crystalline and amorphous lamellae within semicrystalline starch granules [29]. X-ray diffraction analysis distinguishes A-, B-, C-, and V-type crystalline polymorphs, whose occurrence depends on botanical origin, molecular organization, and processing history. These polymorphs differ in their responses to water, thermal treatment, and enzymatic hydrolysis [30].

At a higher structural level, starch occurs as micrometer-scale granules whose size, surface porosity, internal channels, and structural defects influence enzyme penetration and substrate accessibility [31]. This hierarchical architecture regulates degradation through several mechanisms: dense granular surfaces restrict initial enzyme adsorption, highly ordered crystalline regions limit the accessibility of glycosidic bonds, and compact double-helical packing reduces the rate of hydrolytic cleavage [32,33]. The amylose-to-amylopectin ratio is also an important determinant of degradation behavior. High-amylose starches tend to develop more ordered structures and generally exhibit greater resistance to enzymatic hydrolysis than waxy starches [34,35]. The chain-length distribution of amylopectin is similarly important because longer branch chains more readily participate in double-helix formation, thereby increasing crystalline stability and retarding enzymatic degradation [36].

Quantitative measurements also illustrate variation among starch sources. In one comparative study, the measured amylose contents were 22.1% for wheat, 19.8% for normal maize, 17.9% for potato, and 18.7% for tapioca starch, compared with 2.8% for waxy maize. In the same study, the peak gelatinization temperatures were 58.6, 68.7, 61.4, 65.9, and 69.0 °C, respectively. These are values for the samples and test conditions examined, rather than universal ranges for each botanical source [37].

**Figure 1:**
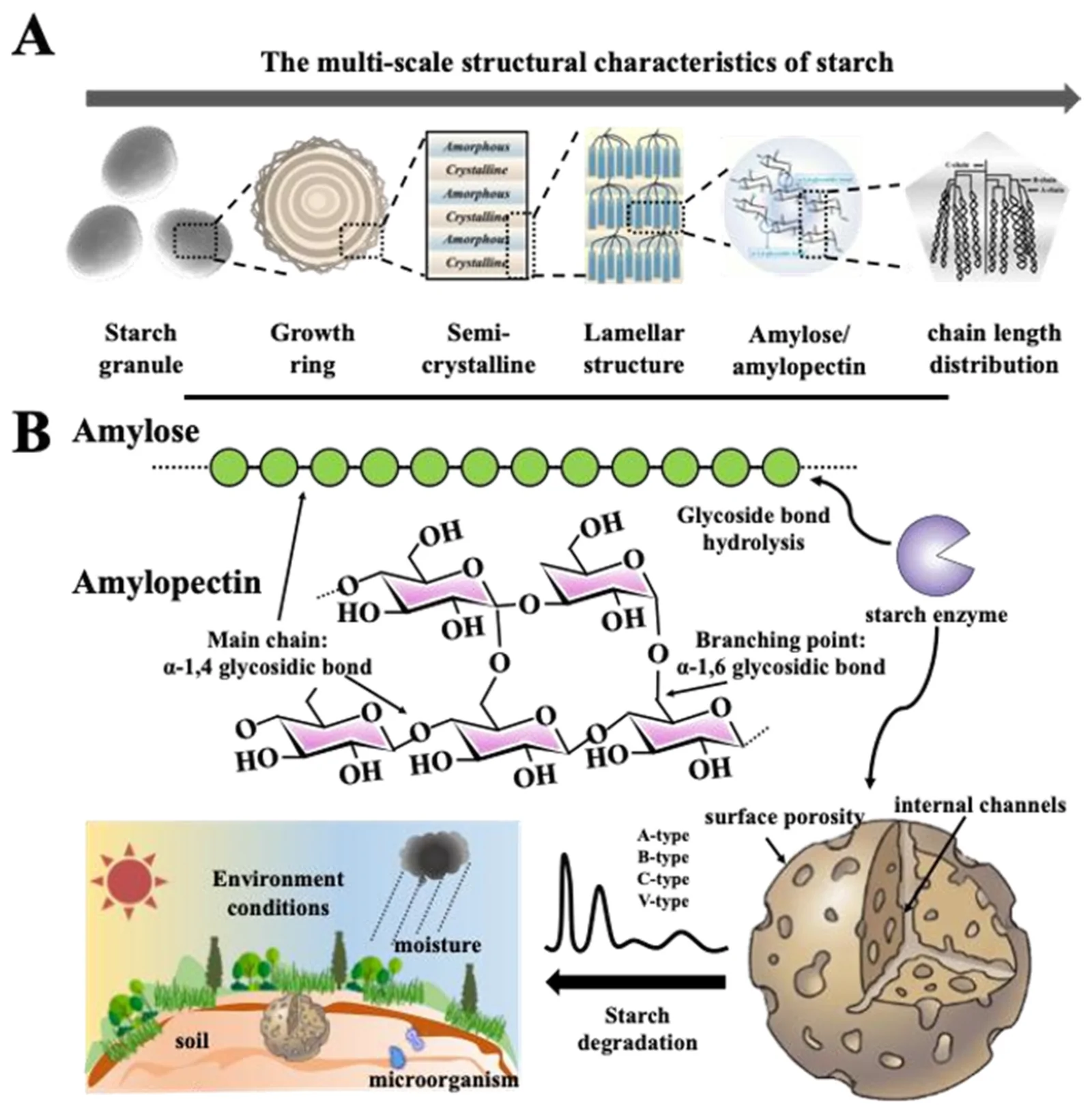
Schematic illustration of the multi-scale structure of starch (**A**) and the enzymatic hydrolysis of amylose/amylopectin coupled with granule degradation (**B**).

In summary, the biodegradability of starch is enabled by enzyme-susceptible glycosidic linkages, whereas its degradation kinetics are regulated by multiscale structural features ranging from molecular conformation to granular morphology (Figure 1). Understanding these structure–property relationships provides a theoretical foundation for analyzing degradation kinetics, designing nanomodification strategies, and achieving more precise control over the degradation behavior of starch-based packaging materials.

### 2.2. Inherent Advantages and Limitations of Starch as a Biodegradable Packaging Material

The principal advantages of starch as a biodegradable packaging material arise from its renewable origin, wide availability, and industrial accessibility. Starch is one of the most abundant natural biopolymers and can be obtained at an industrial scale from agricultural sources such as corn, potato, cassava, and wheat, providing a relatively stable and renewable feedstock for packaging production [38]. Its established extraction and processing infrastructure also offers potential economic advantages compared with many other biodegradable polymers [39]. Under suitable biologically active conditions, starch can be hydrolyzed and metabolized by microorganisms, although its biodegradation rate and degree of mineralization depend strongly on the material formulation and disposal environment [40,41]. Native starch is generally considered biocompatible, while its favorable film-forming properties enable its conversion into packaging films using established techniques such as solution casting, extrusion, and compression molding [42]. These characteristics make starch an important base material for the development of green packaging systems.

Nevertheless, the intrinsic properties of starch also impose substantial limitations on its use in high-performance packaging. Hydrophilicity is one of its most significant disadvantages because the numerous hydroxyl groups along starch chains promote water absorption, swelling, and moisture-induced plasticization. These effects may cause material softening, dimensional instability, and considerable deterioration of barrier performance [43]. Moisture sensitivity not only compromises packaging stability during use but also complicates the controlled regulation of degradation behavior [44]. In addition, unmodified starch films are generally brittle and exhibit limited ductility, relatively low elongation at break, and insufficient mechanical resistance for demanding transportation and storage conditions [45,46].

**Figure 2:**
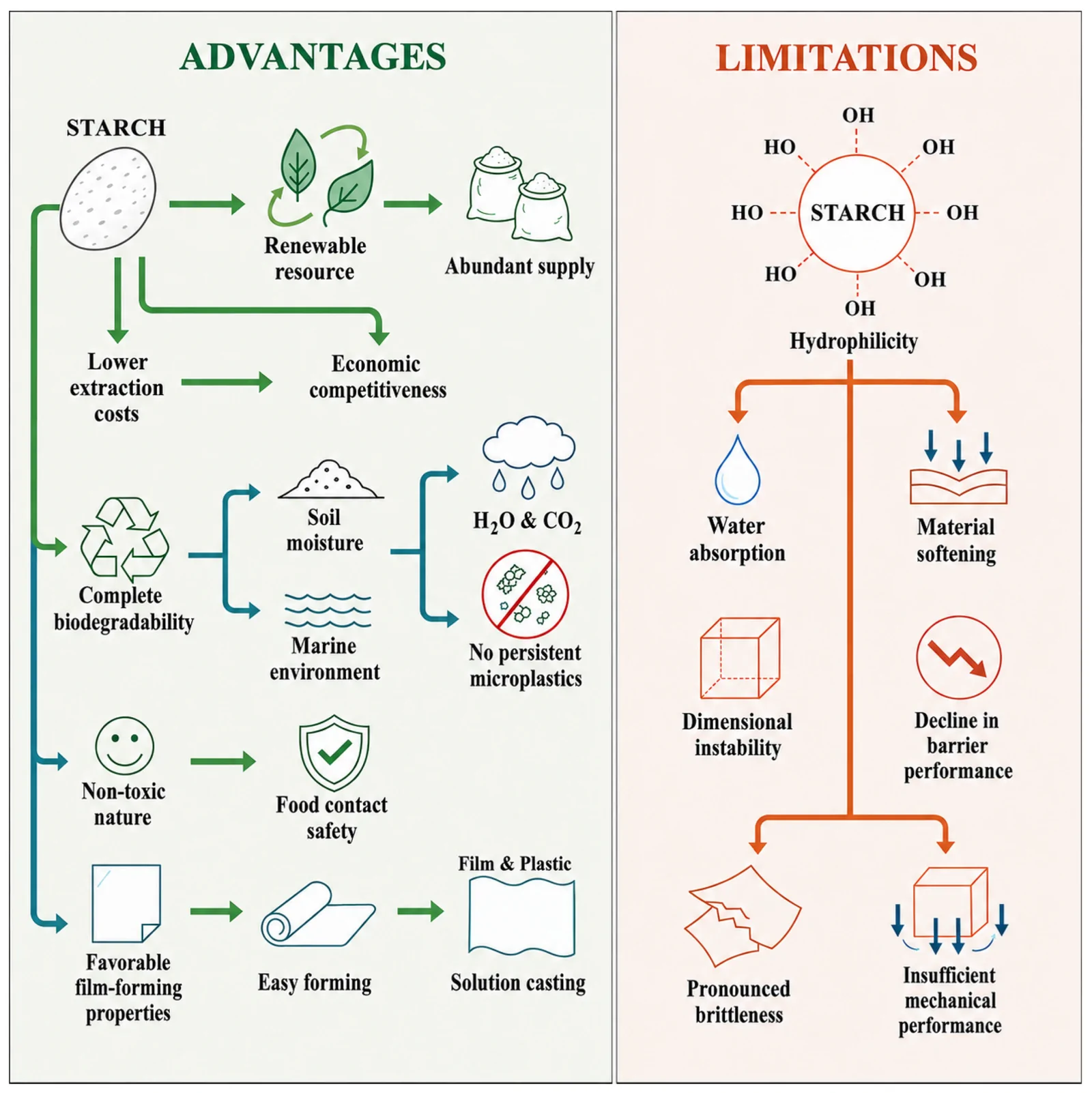
Schematic diagram of the advantages and limitations of starch as a packaging material.

A further challenge is the limited controllability of degradation kinetics. The degradation rate of starch-based materials is highly sensitive to temperature, humidity, microbial activity, oxygen availability, material thickness, crystallinity, and formulation. Consequently, similar materials may exhibit substantially different degradation times under different disposal conditions [47]. This variability complicates the achievement of an optimal balance between stability during storage and use and rapid degradation after disposal. These limitations provide the principal motivation for modifying starch-based packaging materials. Among them, the regulation of degradation kinetics is particularly important because it directly determines performance throughout the material life cycle, from functional packaging to environmentally compatible end-of-life treatment [48,49] (Figure 2).

This need also creates an important opportunity for nanomodification. The incorporation of functional nanofillers can improve mechanical strength, water vapor barrier performance, thermal stability, and interfacial cohesion while simultaneously altering crystallinity, molecular mobility, moisture transport, and enzyme accessibility. Nanomodification therefore provides a potential pathway for addressing the interconnected limitations of hydrophilicity, inadequate mechanical performance, and poorly controlled degradation. However, the effects of nanofillers must be carefully regulated to avoid excessive resistance to biodegradation or unintended environmental impacts.

### 2.3. Role of Starch in Biodegradable Packaging Systems

Starch performs several core functions in biodegradable packaging systems. As a matrix-forming material, it can create the continuous phase of films and coatings, thereby providing basic structural integrity and mechanical support [50,51]. In biodegradable blends, starch may also function as a degradation-initiating component. Preferential hydrolysis and microbial consumption of the starch fraction can generate pores and channels within the material, increase the available surface area, and facilitate water penetration and microbial access to other biodegradable components [52,53]. The concentration, distribution, and structural organization of starch can therefore influence the overall disintegration and degradation behavior of blended packaging materials.

Starch also exhibits synergistic interactions with other polymeric and nanoscale components. When blended with compatible bio-based polymers, starch can reduce feedstock costs and modify water uptake, interfacial properties, and microbial accessibility within the resulting material [54,55,56]. When combined with nanomaterials, the hydroxyl groups of starch can serve as interaction or anchoring sites for nanofillers through hydrogen bonding, electrostatic attraction, or covalent bonding. Conversely, nanofillers may alter starch crystallization, molecular-chain mobility, free volume, and moisture transport, thereby gene-rating synergistic reinforcement and modifying degradation behavior [57]. These bidirectional interactions provide the theoretical basis for the development of targeted nanomodification strategies.

Starch should therefore not be regarded merely as a passive filler in biodegradable blends. Its reactive functional groups participate directly in interactions with plasticizers, polymers, crosslinking agents, and nanofillers, while its structural organization and degradation behavior can be modified through formulation and processing. This responsiveness positions starch as a key component connecting material design, functional performance, and environmental degradation. Clarifying its role in packaging systems is therefore essential for understanding subsequent nanomodification strategies and their effects on degradation control.

## 3. Degradation Mechanisms and Kinetics of Starch-Based Biodegradable Packaging Materials

### 3.1. Degradation Mechanisms

The environmental degradation of starch-based packaging materials is a complex process governed by interacting biological, physical, and chemical factors. These degradation pathways frequently occur simultaneously and may reinforce or inhibit one another, ultimately determining the disintegration, biodegradation, and environmental fate of the material (Table 1).

Biodegradation is the principal degradation pathway for starch-based materials. Microorganisms secrete extracellular enzymes that act on starch molecular chains. α-Amylase randomly cleaves internal α-(1 → 4) glycosidic bonds, generating shorter oligosaccharides and dextrins, whereas β-amylase progressively hydrolyzes α-(1 → 4) bonds from non-reducing chain ends and releases maltose units. Debranching enzymes, including isoamylase and pullulanase, cleave α-(1 → 6) branch points and facilitate further hydrolysis of the amylopectin structure [58,59]. The resulting low-molecular-weight products can be assimilated by microorganisms and subsequently converted into carbon dioxide, water, biomass, and, under anaerobic conditions, other metabolic products.

The relative susceptibility of amylose and amylopectin to enzymatic degradation depends not only on their molecular structures but also on crystallinity, molecular packing, granule organization, and enzyme accessibility. The predominantly linear structure of amylose provides extended α-(1 → 4)-linked regions, whereas the branched structure of amylopectin requires coordinated action by endo-acting, exo-acting, and debranching enzymes [60]. In packaging systems containing starch and cellulose derivatives, cellulases do not directly hydrolyze starch but may degrade the cellulose fraction, weaken the composite structure, and facilitate water penetration and enzymatic access to the starch phase [61,62].

Physical factors strongly influence the rate and extent of biodegradation. Temperature affects microbial activity, enzyme kinetics, water diffusion, and polymer-chain mobility. Consequently, starch-based materials generally degrade more rapidly under thermophilic composting conditions than under ambient soil or aquatic conditions [63,64]. Moisture is another critical parameter because water penetrates the starch matrix, weakens intermolecular hydrogen bonding, acts as a plasticizer, and reduces the glass-transition temperature. The resulting transition toward a more mobile polymer state increases swelling, chain mobility, and enzyme accessibility [65].

Ultraviolet irradiation may induce photo-oxidative reactions within starch-based materials, particularly when photosensitive additives or oxidation-prone blend components are present. These reactions can promote chain scission and generate carbonyl and carboxyl groups, thereby reducing molecular weight and increasing surface hydrophilicity. Surface oxidation and cracking may subsequently facilitate microbial colonization and enzymatic hydrolysis [66]. Nevertheless, the magnitude of this effect depends on formulation, irradiation intensity, exposure time, oxygen availability, and the presence of photosensitizers.

Chemical degradation occurs mainly through hydrolysis and oxidation and generally interacts with biological degradation. During hydrolysis, water molecules promote cleavage of glycosidic bonds, while acidic or alkaline conditions may accelerate chain scission relative to neutral conditions [67]. Oxidative degradation converts hydroxyl groups into carbonyl and carboxyl functionalities, disrupts molecular regularity, and may reduce the degree of crystallinity, thereby increasing water penetration and enzymatic accessibility [68,69]. These pathways do not operate independently. Temperature and humidity promote hydrolysis and oxidation; chemical changes alter the susceptibility of starch chains to enzymes; and microbial metabolism modifies the local pH, oxygen concentration, and redox conditions, further affecting chemical degradation [70].

The relative contribution of each pathway changes dynamically with environmental conditions and material composition. This complexity explains why the degradation behavior of starch-based packaging is difficult to predict using a single parameter or mechanism. At the same time, it provides a theoretical basis for regulating degradation kinetics through the coordinated design of molecular structure, crystallinity, interfaces, additives, and processing conditions.

### 3.2. Degradation Kinetics

Degradation kinetics provides a quantitative framework for describing the degradation behavior of starch-based packaging materials and relating material structure to environmental conditions. Common evaluation parameters include mass loss, molecular-weight reduction, carbon dioxide evolution, degradation percentage, disintegration time, and apparent half-life. Depending on the selected endpoint and degradation mechanism, experimental data may be described using zero-order, first-order, logistic, or other empirical kinetic models.

To make the kinetic descriptions explicit, let M(t) denote the remaining film mass at time t, with M_0_ as the initial mass. A zero-order approximation is M(t) = M_0_ − k_0_t, where k_0_ is the mass-loss rate. For apparent first-order mass loss, M(t) = M_0_e^^−k1t^, where k_1_ is the apparent rate constant and the corresponding half-life is t_1/2_ = \ln(2)/k_1_. When degradation follows a sigmoidal profile, the degraded fraction D(t) may be described by D(t) = D_max_/[1 + e^^−k_L(t−t50)^], where D_max_ is the fitted maximum degraded fraction, k_L_ describes the steepness of the transition, and t_50_ is the time at which the fitted curve reaches D_max_/2. These equations are alternative descriptions of measured endpoints rather than interchangeable measures of complete biodegradation: film mass loss, disintegration, and carbon dioxide evolution represent different processes. Consequently, any comparison of fitted constants between neat starch and nanofiller-containing films should specify the filler loading, film geometry, measured endpoint, and environmental conditions.

**Figure 3:**
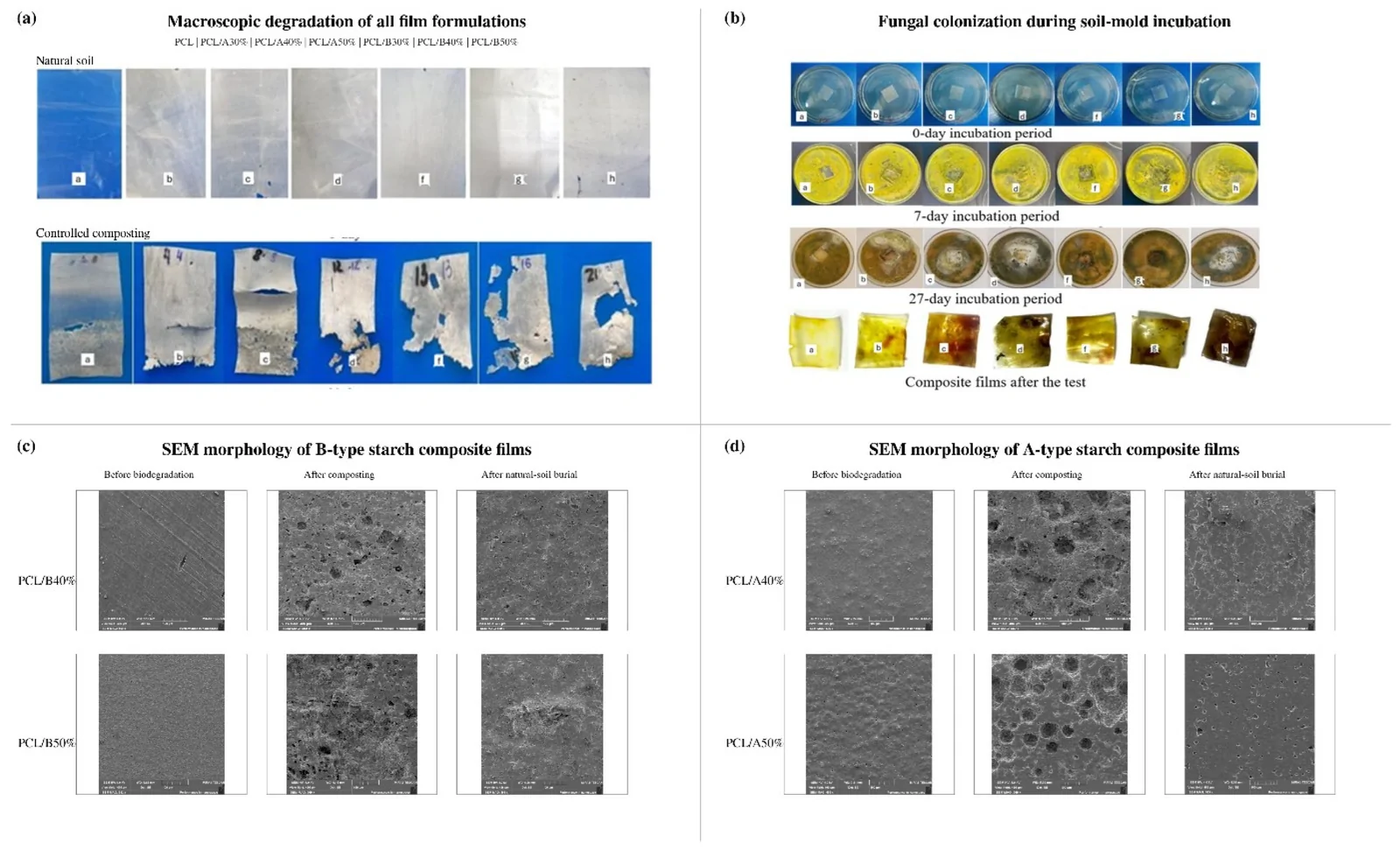
Combined evaluation of film biodegradability and environmental stability. (**a**) Macroscopic degradation of PCL and starch–PCL composite films under natural-soil and controlled-composting conditions. (**b**) Fungal growth on composite films after 0, 7, and 27 days of soil-mold incubation, together with the appearance of the recovered films. (**c**) SEM morphology of PCL/B40% and PCL/B50% films before biodegradation and after degradation in compost and natural soil. (**d**) SEM morphology of PCL/A40% and PCL/A50% films before biodegradation and after degradation in compost and natural soil. Adapted from Ref. [73] under the Creative Commons Attribution 4.0 International (CC BY 4.0) license.

Gravimetric analysis assesses degradation by measuring changes in specimen mass after exposure to soil, compost, water, or other environments. Enzymatic degradation assays use enzymes such as α-amylase and amyloglucosidase under controlled conditions to evaluate the intrinsic susceptibility of the material to hydrolysis. Simulated environmental tests employ controlled chambers, composting reactors, aquatic systems, or soil microcosms to investigate the combined effects of temperature, humidity, oxygen availability, irradiation, and microbial communities [71,72] (Figure 3).

Structural characteristics are intrinsic determinants of starch degradation. The amylose-to-amylopectin ratio, chain-length distribution, crystalline polymorphism, degree of crystallinity, granular morphology, and molecular organization all affect water penetration and enzyme accessibility. High-amylose materials frequently exhibit slower enzymatic hydrolysis than waxy starch systems because of their more compact or ordered molecular organization. Differences among A-, B-, and C-type crystalline structures may also result in distinct hydrolysis profiles, although degradation behavior depends on the combined effects of polymorphism, crystallinity, granule structure, and processing history [74].

Modification strategies may substantially alter degradation kinetics. Cellulose nanocrystals and other high-aspect-ratio fillers can form tortuous diffusion pathways and restrict water and enzyme transport, thereby reducing the initial degradation rate of starch nanocomposites. Conversely, certain metal oxide nanoparticles may promote oxidative chain scission through catalytic or photocatalytic mechanisms under suitable exposure conditions [75,76]. The final effect depends on nanofiller concentration, dispersion, interfacial adhesion, surface chemistry, light exposure, and environmental conditions.

Environmental conditions provide an additional regulatory dimension. Increasing temperature generally accelerates enzymatic and microbial processes within an appropriate biological range, while higher moisture content promotes swelling and molecular mobility. Composting conditions often produce faster degradation than soil or aquatic environments because of their comparatively high temperature, moisture content, microbial density, and aeration [77,78]. In particular, degradation observed under controlled industrial composting conditions should not be extrapolated to ambient soil or aquatic environments. Lower or more variable temperatures, moisture levels, oxygen availability, and microbial activity in these settings may slow disintegration and biodegradation, leaving the material or its components in the environment for longer. Therefore, end-of-life claims should specify the disposal pathway and the conditions under which degradation was measured. Blend composition also influences degradation by changing water uptake, phase morphology, interfacial adhesion, and microbial accessibility. In starch-containing polylactic acid systems, for example, preferential degradation of the starch phase may initially generate pores and channels that promote disintegration of the remaining structure; however, complete biodegradation still depends on the degradability of the non-starch polymer under the selected conditions [79].

Universal degradation times should not be assigned to starch-based materials because the measured rate depends strongly on formulation, specimen geometry, processing history, test method, and disposal environment. Thermoplastic starch may exhibit relatively rapid initial degradation because gelatinization and processing disrupt the native granular and crystalline structures. Subsequent retrogradation, however, may increase structural order and reduce the degradation rate during later stages [80,81]. The incorporation of cellulose nanocrystals may reduce initial water and enzyme penetration and extend the apparent degradation time, demonstrating the possibility of improving service stability through controlled structural reinforcement [82].

Starch-based blends frequently display biphasic degradation behavior. During the initial phase, preferential hydrolysis or microbial consumption of starch produces relatively rapid mass loss and generates pores within the material. During the subsequent phase, the degradation rate may decline as the residual, less degradable polymer fraction becomes dominant [83,84]. These findings demonstrate that degradation kinetics can be adjusted through structural design and formulation. Nevertheless, predictive accuracy, cross-study comparability, and consistency under realistic disposal conditions remain major research challenges (Figure 3).

### 3.3. Strategies for Regulating Degradation Kinetics

**Figure 4:**
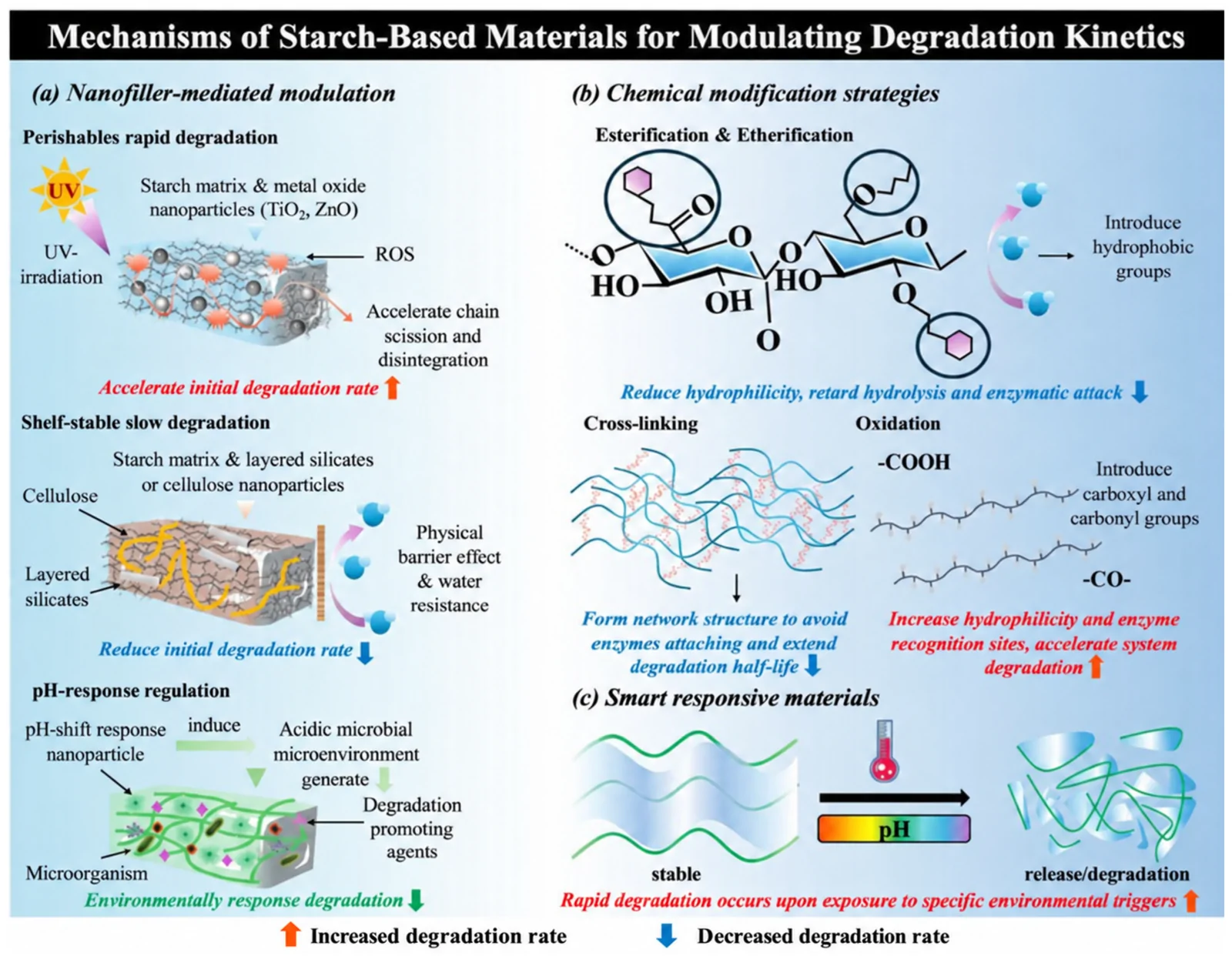
Schematic illustration of degradation kinetics modulation by starch-based materials.

The practical application of starch-based packaging requires degradation kinetics to be aligned with the intended service life. Packaging for products with short distribution periods may permit relatively rapid post-use degradation, whereas packaging for shelf-stable products must retain mechanical integrity and barrier performance for longer periods before degradation begins [85,86]. Effective regulation therefore requires a balance between stability during manufacturing, storage, transportation, and use and rapid biodegradation or disintegration under appropriate end-of-life conditions (Figure 4).

Nanotechnology provides several mechanisms for regulating degradation behavior. Cellulose nanocrystals and layered silicates can form dense or tortuous networks within starch matrices, restricting water penetration and enzyme diffusion and thereby reducing the initial degradation rate [87,88]. In contrast, photocatalytic nanoparticles such as TiO_2_ and ZnO can generate reactive oxygen species under suitable irradiation conditions, promoting oxidative chain scission and surface deterioration [89,90]. Their effectiveness during actual disposal depends on light availability, particle dispersion, surface chemistry, and the surrounding environmental conditions.

The apparent conflict between photocatalytic and antimicrobial effects can be understood in relation to the disposal environment. Under suitable illumination, photoactive nanofillers may generate reactive oxygen species that promote oxidative chain scission at the film surface. In buried soil or poorly illuminated regions of compost, this light-driven process is limited, and degradation depends more strongly on water uptake, enzymatic hydrolysis, and microbial activity. Antimicrobial particles or released ions may affect microorganisms involved in biodegradation and could therefore slow the process under some conditions. However, the overall effect cannot be predicted from photocatalytic or antimicrobial activity alone; it depends on filler type and concentration, particle release, light and oxygen availability, and the microbial community. Thus, results obtained under illuminated conditions should not be directly extrapolated to buried soil or compost.

Responsive nanomaterials offer another potential regulatory approach. pH-responsive nanostructures may alter permeability, interfacial interactions, or active-compound release when exposed to pH changes associated with food deterioration or microbial activity [91,92]. Such systems provide a conceptual basis for environmentally triggered changes in material behavior. However, direct evidence demonstrating reliable, trigger-controlled biodegradation under realistic disposal conditions remains limited and requires further investigation.

Chemical modification provides additional control over starch degradation. Esterification and etherification can introduce less hydrophilic substituents into starch chains, reduce moisture uptake, and retard hydrolytic and enzymatic degradation [93]. Crosslinking creates intermolecular or intramolecular networks that restrict chain mobility, swelling, and enzyme penetration, thereby improving service stability and extending the apparent degradation time [94]. Conversely, controlled oxidation introduces carbonyl and carboxyl groups, increases water affinity, disrupts molecular organization, and may enhance susceptibility to hydrolysis and enzymatic attack [95].

Blending starch with other biodegradable polymers offers a further means of adjusting degradation kinetics. Blend composition, phase continuity, compatibility, and interfacial morphology determine whether starch acts primarily as a structural matrix, a dispersed degradable phase, or a promoter of pore formation and disintegration [79]. The use of compatibilizers and controlled processing conditions is essential because poor interfacial adhesion may cause premature mechanical failure, whereas excessive compatibility or crystallinity may restrict water and enzyme transport.

Smart and stimuli-responsive starch-based materials represent an emerging strategy for achieving service stability followed by accelerated end-of-life transformation. Thermo-responsive, pH-responsive, photo-responsive, or moisture-responsive components may be incorporated to alter permeability, swelling, chain mobility, or degradation susceptibility after exposure to specific environmental triggers [96,97]. However, these systems must be evaluated for trigger reliability, additive safety, scalability, environmental persistence, and compatibility with existing waste-management infrastructure.

Overall, nanomodification, chemical derivatization, blending, and stimuli-responsive design provide complementary approaches for controlling degradation kinetics. Nanomodification is particularly promising because nanofillers can simultaneously influence mechanical strength, barrier properties, crystallinity, water transport, and enzyme accessibility. Nevertheless, successful application requires the degradation benefits to be balanced against nanofiller toxicity, environmental fate, processing complexity, and production cost.

## 4. Advanced Nanomodification Strategies for Starch-Based Biodegradable Packaging Materials

### 4.1. Nanomodification Technologies

The integration of nanomodification technologies provides new opportunities to overcome the performance limitations of starch-based packaging materials. Functional nanofillers can influence the hierarchical organization of starch at the molecular, supramolecular, and interfacial levels, thereby modifying mechanical performance, barrier properties, thermal stability, and degradation behavior. Current research encompasses zero-dimensional nanoparticles, one-dimensional nanofibers and nanocrystals, and two-dimensional nanosheets and nanoplatelets [98].

Cellulose nanofibers and cellulose nanocrystals are among the most widely investigated bio-based nano-reinforcements because of their high aspect ratios, favorable mechanical properties, and chemical affinity for starch matrices. Hydroxyl groups on nanocellulose surfaces can form hydrogen-bonding networks with starch chains, improving interfacial adhesion and restricting molecular mobility. These interactions may increase mechanical strength and alter water transport and enzyme diffusion [99,100]. However, the resulting effects depend strongly on nanocellulose concentration, dispersion, surface chemistry, and the moisture content of the matrix.

Layered silicate nanoplatelets, including montmorillonite, can create tortuous diffusion pathways within starch matrices, increasing the effective transport distance of water vapor and gases and improving barrier performance [101]. Emerging two-dimensional nanomaterials such as MXenes and graphene oxide have high specific surface areas and abundant surface functional groups. At relatively low concentrations, they may form interconnected networks that improve barrier, electrical, mechanical, or antimicrobial functionality [22,102]. Nevertheless, their environmental persistence, migration behavior, and compatibility with biological end-of-life pathways require careful evaluation.

Metallic and metal oxide nanomaterials provide additional functionality. Silver nanoparticles are primarily incorporated for their antimicrobial activity and may inhibit microbial proliferation on packaging surfaces [103]. Photoactive metal oxide nanoparticles may promote reactive-oxygen-species formation under suitable irradiation conditions and thereby accelerate oxidative chain scission [89]. Because antimicrobial nanoparticles may also suppress microorganisms involved in biodegradation, their net effect on environmental degradation must be evaluated under realistic disposal conditions rather than inferred solely from antimicrobial performance.

Nanomodification influences starch-based packaging through three principal mechanisms. First, it can regulate water transport, molecular mobility, and enzyme accessibility. Dense hydrogen-bonding or percolation networks formed by cellulose nanocrystals may reduce water penetration and enzyme diffusion during storage and use, whereas photoactive nanoparticles may promote chain scission after exposure to suitable end-of-life conditions [61,89,100]. This creates the possibility of improving service stability while retaining post-use degradability.

Second, nanofillers may simultaneously improve mechanical and barrier properties. Nanocellulose commonly increases tensile strength, although its effect on elongation at break depends on dispersion, interfacial adhesion, plasticizer content, and filler concentration. Layered silicates can reduce oxygen and water vapor permeability by increasing diffusion-path tortuosity [100,101,102,103,104,105]. These improvements may extend product shelf life and permit reductions in packaging thickness, provided that brittleness and processing stability remain acceptable.

Representative results illustrate the importance of reporting both filler loading and matrix composition. For starch–MXene films, the addition of 7.5 wt% MXene increased tensile strength from 9.9 to 19.1 MPa in the formulation studied by Dong et al. [104]. The same study reported reductions of up to 92.9% in water vapor permeability and 74.0% in oxygen permeability for its modified films. In a separate study using chemically modified corn starch as the matrix, formulations containing 0.1 wt% organically modified montmorillonite and varying glutaraldehyde concentrations were examined; the highest reported tensile strength was 30.22 MPa [103]. These results demonstrate measurable improvements, but their magnitudes depend on filler loading, matrix chemistry, plasticizer or crosslinker content, and test conditions. Changes in elongation at break and degradation behavior should therefore be assessed separately for each formulation.

Third, nanomodification may contribute to life cycle sustainability by enabling material reduction, extending food shelf life, and replacing fossil-derived additives with renewable fillers [104,105]. However, these environmental benefits are not automatic. They must be demonstrated by considering nanofiller production, energy demand, solvent use, migration, ecotoxicity, material recovery, and end-of-life behavior. Table 2 summarizes the classification of nanomaterials, their key mechanisms and performance outcomes, and synergistic and green approaches for starch-based packaging materials.

Despite the substantial progress achieved through nanomodification, several critical challenges remain to be addressed. The dispersion of nanoparticles within the starch matrix constitutes the most fundamental obstacle; nanofillers with high surface energy exhibit a pronounced tendency to aggregate, which not only compromises the reinforcing efficiency but may also introduce stress concentration sites, leading to performance deterioration [124]. Insufficient interfacial compatibility further constrains property enhancement: the thermodynamic incompatibility between hydrophobic nanofillers and hydrophilic starch matrices results in interfacial debonding, necessitating complex surface modification procedures that subsequently escalate material costs [15]. Economic feasibility for large-scale production presents another practical barrier to industrial translation and cleaner production, as most cutting-edge nanomodification research remains confined to laboratory scales with inadequate cost–benefit assessments [125]. In response to these challenges, green nanomodification strategies have garnered increasing attention in recent years. The synthesis of nanoparticles through plant extracts or microbial methods circumvents the generation of toxic byproducts associated with conventional chemical synthesis. Naturally derived polymers such as chitosan and alginate can self-assemble into nanostructures during blending with starch, achieving in situ reinforcement without the incorporation of exogenous nanofillers [126]. The design of multifunctional nanoparticles represents another frontier: the integration of multiple functionalities, including antimicrobial, antioxidant, and pH-responsive properties, into a single nanofiller enables simultaneous matrix reinforcement and the impartation of active packaging capabilities, thereby expanding potential application scenarios [127,128]. These emerging directions offer innovative pathways for overcoming current modification bottlenecks and advancing starch-based packaging materials toward enhanced performance and functionality.

For food-contact applications, improved antimicrobial or barrier performance alone is insufficient to establish the safety of a starch-based nanocomposite. Potential migration of nanofillers or released ions should be evaluated under contact conditions relevant to the intended food, including contact time, temperature, and food composition. Toxicological assessment should consider both the released amount and the form in which a substance migrates. As a regulatory example, the European Union’s framework for food-contact materials requires that constituents must not transfer to food in quantities that endanger human health; under its rules for plastic food-contact materials, substances in nanoform require explicit authorization. The applicability of specific requirements to a starch-based formulation must be assessed according to its material composition and intended market.

### 4.2. Synergistic Modification Technologies

Despite the considerable advancements achieved through single-strategy nanomodification, its inherent limitations have become increasingly apparent when addressing the complex requirements for synergistic optimization of multiple performance attributes in starch-based materials. The integration of complementary modification pathways, encompassing physical–nano, chemical–nano, enzymatic–nano, and blending–nano synergies, offers effective strategies for resolving the intrinsic contradictions among dispersion, interfacial compatibility, and performance regulation.

Physical–-nano synergistic modification, which combines ultrasound-assisted treatment with nanomodification, substantially enhances the dispersion state of nanofillers within starch matrices. Ultrasonic cavitation generates localized high temperature and pressure that disrupt nanoparticle agglomerates while simultaneously promoting the disentanglement of starch molecular chains, thereby establishing favorable conditions for uniform filler distribution [106]. Shear fields during extrusion processing act synergistically with nanofillers to induce orientation of starch chains along the flow direction, yielding anisotropic structures that improve tensile strength relative to nanomodification alone [107]. Modulation of thermal profiles during melt blending optimizes the degree of starch plasticization, enhancing interfacial wetting between nanofillers and the matrix while reducing the density of interfacial defects [108].

Chemical–nano synergistic modification couples chemical derivatization of starch chains with nanofiller incorporation to generate dual reinforcement effects. Esterification or etherification introduces hydrophobic moieties onto molecular chains prior to compounding with cellulose nanocrystals, concurrently addressing excessive hydrophilicity and inadequate interfacial compatibility [109]. Crosslinking establishes network structures between starch molecules, and subsequent introduction of layered silicates anchors nanoplatelets within the crosslinked network, simultaneously restricting filler migration and agglomeration while further enhancing network compactness, leading to improved water vapor barrier performance [110]. Graft copolymerization covalently attaches polymer chains to nanofiller surfaces, creating brush-like structures that substantially enhance interfacial adhesion strength, maintaining degradation performance while achieving improved tensile properties [111].

Enzymatic–nano synergistic modification integrates enzymatic treatment with nanomodification and provides a biocatalytic pathway for regulating starch multiscale structure. Selective hydrolysis of amylopectin branching points by amylases adjusts the amylose-to-amylopectin ratio prior to compounding with nanocellulose, enhancing filler reinforcement efficiency due to improved compatibility between the more ordered molecular chains and the nanofillers [112]. Transglycosidase catalyzes the rearrangement of starch molecular chains, altering their chain length distribution and branching structure; nanocomposites constructed from the modified starch enable effective modulation of enzymatic susceptibility, thereby allowing the degradation half-life to be tuned across a broad range [113].

Blending–nano synergistic modification involves the incorporation of nanofillers into blends of starch with other biobased polyesters, establishing ternary composite systems that enable multilevel regulation of degradation behavior. In starch/polylactic acid/cellulose nanocrystal ternary systems, polylactic acid provides initial structural stability, the starch component functions as a degradation trigger unit, and cellulose nanocrystals modulate water vapor permeation and subsequent disintegration kinetics. This tripartite synergy yields degradation profiles in soil environments characterized by a desirable delayed–accelerated–completed three-stage pattern [114]. In starch/montmorillonite composites, nanoplatelets preferentially localize at interfacial regions, simultaneously improving interfacial adhesion between immiscible components and forming localized barrier layers that delay degradation onset, offering a viable approach for matching medium-to-long-shelf-life food packaging requirements [101].

The core advantages of synergistic modification lie in transcending the trade-offs inherent in single-modification approaches. Physical–nano synergy addresses dispersion issues without introducing chemical reagents, preserving the green attributes of the material. Chemical–nano synergy locks in modification effects through covalent bonding while enhancing performance, improving long-term stability. Enzymatic–nano synergy leverages biocatalytic specificity to achieve targeted regulation of the fine starch structure. Blending–nano synergy enables temporal decoupling and precise programming of degradation behavior through functional compartmentalization of multiple components. Collectively, these synergistic pathways converge on a common objective: achieving simultaneous optimization of mechanical properties, barrier performance, and degradation kinetics while preserving the intrinsic biodegradability of starch, thereby delivering customizable material solutions tailored to diverse application scenarios.

### 4.3. Green Nanomodification Technologies

The emergence of green nanomodification technologies addresses the core imperative of life-cycle sustainability in starch-based packaging materials, as these approaches utilize renewable feedstocks, green solvents, and low-energy processes to achieve performance enhancement while reducing the environmental footprint of the nanomodification stage. Agricultural waste streams show great potential as precursors for nanofiller production: cellulose-rich residues such as rice husks, wheat straw, and corn stover can be used to extract cellulose nanocrystals and nanofibers via acid hydrolysis or mechanical treatment, establishing a waste-to-resource circulation paradigm [115]; bagasse-derived cellulose nanocrystals prepared by bleaching and acid hydrolysis provide reinforcement efficiency comparable to wood pulp-based counterparts in starch matrices, enhancing mechanical and barrier properties with significantly lower feedstock costs [116]; and tropical agricultural residues including banana pseudostems and pineapple leaves produce high-aspect-ratio cellulose nanofibers, offering localized green modification routes for starch packaging industries in tropical regions [117]. Green solvent-assisted nanomodification overcomes the environmental and safety issues of conventional organic solvents: ionic liquids act as designable green solvents that dissolve starch and disperse nanofillers simultaneously, allowing homogeneous modification and solvent recovery through antisolvent precipitation with good recyclability over multiple cycles [118]; aqueous systems using high-pressure homogenization and microfluidization enable efficient nanofiller dispersion without organic solvents, supporting the scalable preparation of cellulose nanocrystal–starch composite films [119]; and supercritical carbon dioxide-assisted modification uniformly loads nanofillers into porous starch structures while achieving sterilization and drying, simplifying the overall process flow [120]. In addition to cellulose, various biobased nanomodifiers have gained increasing attention: chitosan nanoparticles fabricated by ionic gelation exhibit intrinsic antimicrobial activity, delaying microbially mediated degradation in starch matrices with complete biodegradability and no secondary pollution [121]; starch nanocrystals obtained by acid or enzymatic hydrolysis show excellent interfacial compatibility with the starch matrix due to chemical homology, allowing uniform dispersion without surface modification to form all-starch nanocomposites [122]; and lignin nanoparticles derived from paper industry byproducts provide antioxidant activity through phenolic hydroxyl groups, retarding premature aging caused by oxidative degradation [123]. The integration of these green technologies delivers multifaceted core advantages: agricultural byproducts reduce feedstock costs, while green solvent recycling lowers reagent consumption, leading to overall lower production costs compared with conventional nanomodification; eliminating organic solvents reduces volatile organic compound emissions, and life-cycle assessments confirm a lower carbon footprint for the green nanomodification stage; furthermore, biobased nanofillers degrade simultaneously with starch without persistent nanoscale contaminants, and ecotoxicological evaluations reveal lower inhibitory effects on soil microbial communities and aquatic organisms relative to conventional metal-based nanoparticles, thus comprehensively enhancing the life-cycle sustainability of starch-based packaging throughout its life cycle.

## 5. Environmental Impact Assessment of Starch-Based Biodegradable Packaging Materials

### 5.1. Environmental Impact Evaluation Indicators and Methods

Life-cycle assessment (LCA) should be framed in accordance with the principles and framework of ISO 14040 [129] and the requirements and guidelines of ISO 14044 [130]. The goal and scope definition should specify the functional unit, system boundary, data sources, and impact categories before results are compared. For packaging, a mass-based functional unit, such as 1 kg of film, can describe material production, whereas a function-based unit should additionally reflect the amount of food protected and the required shelf life. A cradle-to-gate boundary covers raw-material production and film manufacture; a cradle-to-grave boundary also includes use and end-of-life treatment. Global warming potential, fossil-resource use, eutrophication, acidification, water use, and land use should be reported separately, because an advantage in one category does not establish an overall environmental advantage.

The life-cycle sustainability of starch-based biodegradable packaging materials requires systematic assessment through well-established frameworks including life-cycle assessment, carbon-footprint analysis, and degradation product safety evaluation, which collectively quantify environmental performance across the full life cycle: life-cycle assessment evaluates impacts from raw material acquisition, manufacturing, and use to end-of-life disposal across key categories such as global warming potential, fossil resource depletion, and ecosystem toxicity, while carbon-footprint analysis focuses on greenhouse gas emissions by distinguishing biogenic carbon fixed during crop growth from fossil-derived carbon emissions, with comparative studies confirming that starch-based materials present lower global warming potential and fossil resource consumption than petroleum-based alternatives despite significant contributions to eutrophication and acidification from fertilizer application in the agricultural cultivation stage [131,132]; comprehensive environmental safety evaluation extends beyond verifying complete mineralization to CO_2_ and water by requiring ecotoxicological testing of intermediate degradation products using multi-trophic indicator organisms (bacteria, microalgae, aquatic invertebrates, and higher plants) to assess acute and chronic toxicity, with special attention to the fate and toxicity of nanoscale components potentially released during matrix breakdown for nanofiller-containing starch-based materials [133,134]; core evaluation indicators include energy consumption (integrating life cycle direct inputs and renewable energy share), carbon emissions (capturing direct and embodied releases with separate biogenic carbon accounting to prevent double-counting), degradation product toxicity (combining hazard identification and exposure assessment for human and ecological risk characterization), and resource utilization efficiency (quantifying functional output-to-input ratio for circular economy analysis) [135,136]; furthermore, methodological frontiers feature dynamic life-cycle assessment that incorporates temporal emission heterogeneity to improve the accuracy of long-term release characterization during degradation and regionalized approaches that account for spatial variations in agricultural practices and waste management infrastructure, yet persistent challenges remain including the dependence of biogenic carbon neutrality assumptions on land management practices, the characterization of degradation behavior in uncontrolled environments, and the establishment of consensus methodologies for comparing degradable and non-degradable materials across diverse end-of-life scenarios.

### 5.2. Environmental Impact of Starch-Based Packaging in the Whole Life Cycle

**Figure 5:**
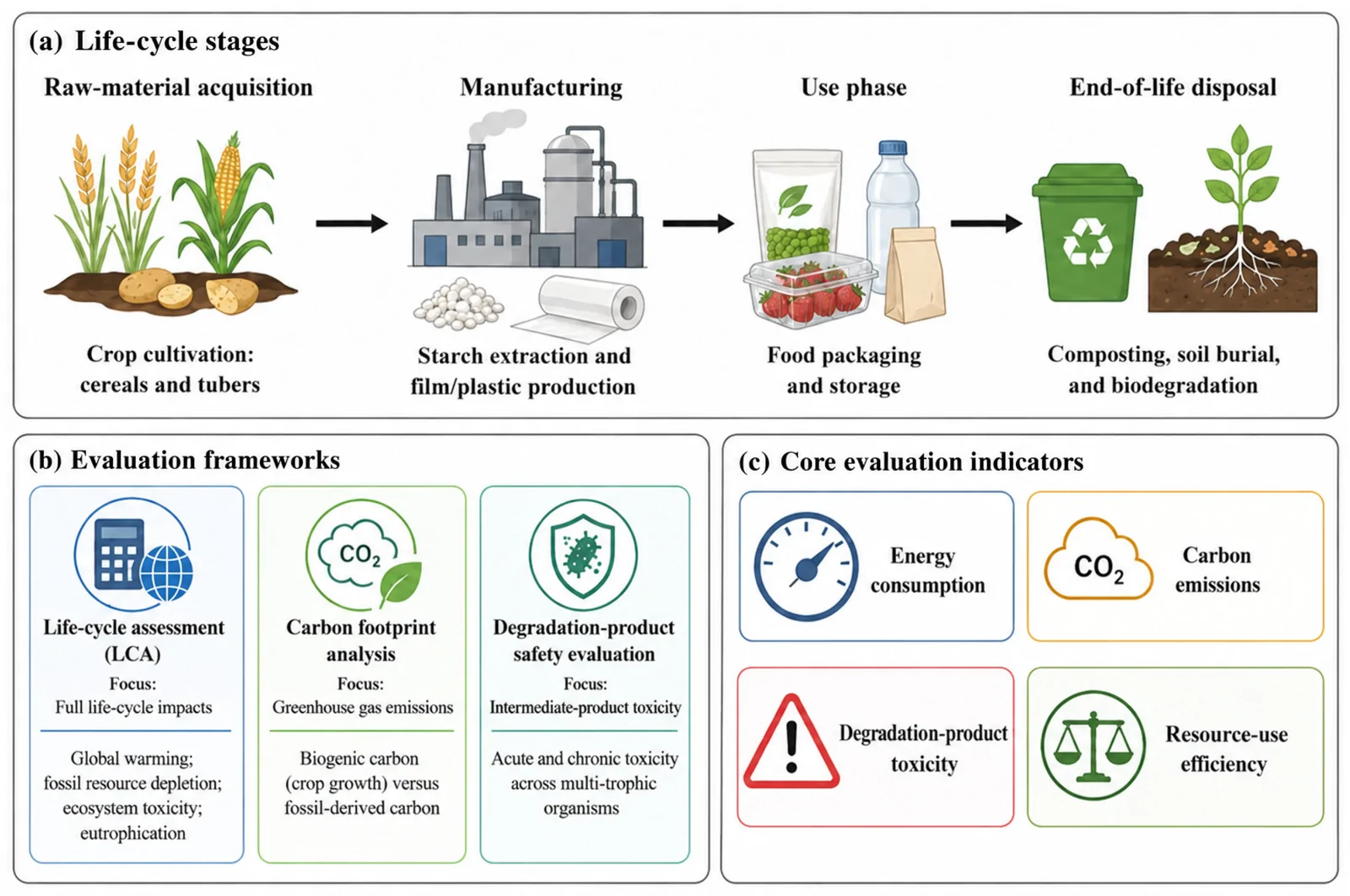
Schematic illustration of the life-cycle environmental impact patterns and key assessment indicators of starch-based packaging throughout its entire life cycle.

The environmental sustainability of starch-based packaging materials should be systematically assessed within a full life cycle framework (Figure 5). In the raw material production stage, some reported comparisons indicate a lower carbon footprint for starch-based materials than polyethylene under the assumptions examined; however, the results should not be generalized across packaging functions and end-of-life scenarios [137]. During the modification and processing stage, while nanomodification improves material performance, the production of conventional nanofillers is highly energy-intensive; green nanomodification technologies, such as agricultural waste-derived nanocrystals and water-based dispersion processes, can substantially lower processing energy consumption and limit the increase in carbon footprint during modification to a low level [138]. In the service stage, the barrier network constructed by nanomodification extends food shelf life and reduces indirect carbon emissions from food waste, with the emission reduction effect far outweighing the environmental burden of the packaging material itself. At the end-of-life stage, the starch matrix may biodegrade under suitable composting conditions. However, this does not mean that incorporated nanofillers also degrade or disappear. Anaerobic landfill conditions may additionally lead to methane generation [98]. Comparisons between starch-based packaging and commodity plastics must use the same functional unit, such as the packaging required to protect a defined quantity of food for a specified shelf life. They should account for feedstock cultivation, land and water use, film production, food loss, collection, recycling or composting, and final disposal. Although starch-based materials may reduce fossil-resource use, their overall environmental advantage cannot be assumed: starch feedstock and processing inputs are required for each production cycle, while the burdens of collected and recycled plastics, including PET, may be distributed across multiple material-use cycles. The result therefore depends on collection rates, recycling yields, the number of effective cycles, packaging performance, and the selected end-of-life scenario. The available studies do not provide a sufficiently consistent basis for a single quantitative comparison across these scenarios; future LCAs should report results per equivalent packaging function and include sensitivity analyses for recycling rates and starch feedstock and processing inputs [139].

When the starch matrix breaks down, persistent nanofillers such as TiO_2_, silver nanoparticles, and graphene oxide may be released into compost, soil, or water. Depending on the material and environmental conditions, these fillers may remain as particles, aggregate, dissolve, or undergo chemical transformation. Therefore, film mass loss alone cannot demonstrate the environmental safety of the complete composite. Future end-of-life studies should separately assess starch degradation, nanofiller release and transformation, and potential exposure of organisms [133,134].

The long-term ecological implications of nanofiller release require assessment beyond short-term film disintegration or acute toxicity tests. Following starch-matrix degradation, released particles and dissolved species may be retained in soil, transported with pore water, or transformed through aggregation, dissolution, and interactions with soil organic matter. For antimicrobial fillers in particular, potential effects on soil bacteria and fungi involved in organic-matter decomposition should be assessed under environmentally relevant exposure concentrations. Repeated inputs and chronic exposure may be more informative than a single short-term test. Future studies should therefore compare an unfilled starch-film control with nanocomposite films and measure particle or ion release, changes in microbial community composition, and indicators of microbial activity over time. These are research needs rather than evidence that every released nanofiller causes the same effect.

Representative LCA results help illustrate the magnitude and variability of these impacts. In a study using 1 kg of product at the factory gate as the functional unit, the net cradle-to-gate global warming potential was 1.37 kg CO_2_ eq for thermoplastic starch pellets and 3.55 kg CO_2_ eq for starch-based packaging films containing additional polyester components. The difference reflects the effects of formulation and film production. These values should not be interpreted as a universal advantage over PET or other conventional plastics, because meaningful comparisons also require equivalent packaging performance, consistent system boundaries, and consideration of collection and recycling rates. Further studies should report energy demand, water use, and agricultural impacts alongside global warming potential under clearly defined end-of-life scenarios [137].

### 5.3. Optimization Strategies for Reducing Environmental Impact

To address the environmental vulnerabilities across the life cycle of starch-based packaging materials, recent studies have proposed multiple actionable optimization strategies. Regarding feedstock greening, the recovery of starch from food processing byproducts such as potato peels, rice bran, and corn gluten avoids competition for land resources with food crops while reducing the environmental burden associated with agricultural inputs. In terms of low-carbon modification processes, water-based physical dispersion techniques replace organic solvent systems, achieving uniform nanofiller distribution through ultrasound assistance or high-pressure homogenization while eliminating volatile organic compound emissions [140]. The development of room-temperature or low-temperature processing conditions significantly reduces energy consumption during the modification stage. The integration of continuous extrusion processing with online nanocomposite fabrication shortens the process flow and minimizes energy losses across intermediate steps [141]. Concerning harmless regulation of degradation products, the selection of biobased nanofillers such as cellulose nanocrystals, chitosan nanoparticles, and starch nanocrystals as substitutes for metallic or synthetic nanoparticles ensures that no persistent nanoscale contaminants remain after material degradation. Nanomodulation enables the controlled initiation and rate of degradation, preventing methane release or microplastic formation under improper disposal scenarios such as landfilling or uncontrolled environmental release [21,142]. The incorporation of functional components that promote degradation, including natural antioxidants and enzyme-encapsulated nanoparticles, accelerates end-of-life degradation while maintaining service stability [143]. Collectively, these optimization strategies converge on the common objective of reducing the environmental burden across the entire chain from feedstock sourcing and processing to end-of-life disposal, while preserving material performance, thereby advancing starch-based packaging materials from mere biodegradability toward genuine, verifiable full life-cycle environmental benefits.

### 5.4. Techno-Economic Feasibility and Comparison with Conventional Plastics

The low price and broad availability of starch do not, by themselves, establish the cost competitiveness of modified starch bio-composites. Their production cost also depends on plasticizers, biodegradable polyesters, nanofillers, compatibilizers, energy consumption, processing yield, and the equipment required to produce films with consistent properties. Feedstock scalability must therefore be assessed at the level of the complete formulation rather than starch alone. Although agricultural and food-processing residues may provide alternative starch sources, their collection, extraction, purification, and variable composition can introduce additional costs.

The choice of manufacturing route is a major economic consideration. Solution casting is useful for laboratory evaluation, but solvent removal and drying increase processing time and energy demand. Melt extrusion and film blowing are more relevant to continuous production, although starch moisture content, plasticization, and filler dispersion require careful control. Additional drying, separate compounding steps, or surface treatment of nanofillers can increase both energy use and equipment costs. A study comparing one-step and two-step processing of thermoplastic starch/polyester blends demonstrated that process configuration affects film quality and the number of manufacturing operations required [144]. These findings indicate that a formulation with inexpensive raw materials may still be costly if it requires multiple processing stages or produces substantial off-specification film.

Available cost estimates also depend strongly on formulation and study assumptions. For example, a techno-economic study of PBAT–starch–nanoclay films reported estimated production costs of ₹343.07–360.62 kg^−1^ across its tested formulations; increasing starch substitution reduced the study’s estimated variable costs [145]. These figures describe that particular production scenario and should not be treated as a general price for starch-based packaging.

Comparison with LDPE and PET requires equivalent packaging functions and consistent system boundaries. LDPE is a relevant benchmark for flexible films, whereas PET is more appropriate where its strength, clarity, or barrier function matches the intended application. A comparison based solely on resin price per kilogram may be misleading if the candidate materials require different film thicknesses, processing steps, or storage conditions to protect the same quantity of food. Future techno-economic assessments should therefore report raw-material and additive costs, electricity and thermal energy consumption, equipment and labor costs, production rate, yield, and cost per functional packaging unit. They should also test how these results change with plant capacity and feedstock price. At present, the limited number of directly comparable studies prevents a general conclusion that modified starch bio-composites are cost-competitive with LDPE or PET across food-packaging applications.

### 5.5. Comparison with Other Bio-Based Packaging Materials

Starch-based packaging offers abundant feedstocks, relatively low raw-material costs, and formulation flexibility, but its hydrophilicity can limit mechanical stability and moisture-barrier performance under humid conditions. Compared with starch, polylactic acid (PLA) generally offers greater compatibility with conventional melt-processing equipment and better moisture resistance, although its production and end-of-life performance depend on the formulation and the availability of suitable disposal infrastructure [1]. Cellulose-based packaging can provide useful mechanical reinforcement and oxygen-barrier performance; however, its moisture sensitivity and the additional processing needed to produce films or coatings must also be considered [122]. Chitosan-based materials are attractive for active packaging because of their film-forming and antimicrobial properties, while feedstock availability, formulation cost, and performance under specific food-contact conditions may constrain their use [128]. These materials can also be combined with starch to address particular performance limitations. No single bio-based material is optimal for every food-packaging application: comparisons should use equivalent packaging functions and report mechanical strength, water-vapor and oxygen barriers, food-preservation performance, processing requirements, and end-of-life conditions under comparable test settings [146,147].

## 6. Application of Starch-Based Biodegradable Packaging Materials

### 6.1. Application in Packaging

The suitability of starch-based nanocomposites for food packaging should be evaluated across multiple performance dimensions rather than by improvement in a single property. Mechanical strength and elongation, water vapor and oxygen barrier properties, food preservation performance, processability, and end-of-life behavior should be compared under clearly specified test conditions. Nanofiller type, loading, dispersion, and interfacial interactions can produce trade-offs among these properties; therefore, the preferred formulation depends on the requirements of the packaged food and its distribution conditions [146,147].

The application suitability of starch-based packaging materials for different food categories is closely related to their degradation kinetic characteristics. Fresh fruit and vegetable packaging requires the material to maintain structural integrity during the appropriate moisture and gas permeability preservation period, with the degradation initiation time needing to match the shelf life (approximately 7–21 days) to avoid premature degradation leading to packaging failure. Nanomodification delays water molecule permeation through the incorporation of cellulose nanocrystals or layered silicates, extending the degradation half-life to align with the preservation period of fresh produce, and its effectiveness has been validated in short-term preservation packaging for fruits and vegetables [148,149]. Meat and seafood products demand high barrier and antimicrobial properties from packaging, along with degradation behavior that adapts to cold-chain environments (0–4 °C); the incorporation of chitosan nanoparticles or silver nanoparticles imparts antimicrobial functionality, inhibiting spoilage microorganisms, while the dense barrier layer constructed through nanomodification reduces the oxygen transmission rate and delays lipid oxidation. Degradation kinetic studies indicate that such materials exhibit significantly reduced degradation rates under refrigerated conditions, meeting the requirements of cold-chain transportation cycles [150,151]. Dairy product packaging demands stringent moisture barrier properties and resistance to acidic environments (yogurt, cheese). Starch-based films incorporated with nanomaterials can achieve a reduction in water vapor transmission rate; meanwhile, the introduction of pH-responsive nanoparticles enables a packaging color response or transition in degradation behavior triggered by pH changes resulting from product spoilage, thereby realizing freshness indication functionality [92,152]. Processed food packaging (e.g., biscuits, instant noodles) requires extended shelf life (3–12 months), imposing higher demands on degradation rate control. Composite systems based on high-amylose starch blended with polylactic acid enable the extension of degradation initiation time by adjusting the ratio of starch to polyester, while maintaining the mechanical stability and barrier performance of the packaging [153].

Representative food tests provide a clearer indication of application performance. For example, a maize starch–PVA nanocomposite film was reported to preserve button mushrooms during refrigerated storage for up to 14 days at 4 °C [86]. In intelligent packaging, a porous starch composite coated with anthocyanins and carboxymethyl cellulose exhibited a visible pH response over the range of 6–11.5, illustrating its potential for monitoring seafood freshness [150]. These results describe specific formulations and test conditions and should not be generalized to all starch-based packaging systems.

Food-contact conditions also affect the choice of starch-based packaging. High-moisture foods may promote water uptake and swelling of the starch matrix, weakening its mechanical integrity and moisture barrier during storage. For high-fat foods, the assessment should additionally consider lipid oxidation, interactions with incorporated active compounds, and the possibility that those compounds migrate into the food. Packaging performance should therefore be verified using the intended food or a suitable food simulant under the relevant storage temperature, humidity, and contact duration.

Moreover, intelligent responsive starch-based packaging has emerged as a cutting-edge research hotspot. pH-responsive packaging incorporates natural pigments (anthocyanins, curcumin) loaded into nanocarriers and composited with starch matrices, which exhibit color changes in response to pH variations caused by food spoilage, enabling real-time freshness indication. Meanwhile, nanomodulation regulates the sustained-release behavior of pigments, prolonging the timeliness of the indicating function [152,153,154]. Temperature-responsive packaging introduces thermosensitive polymer-grafted nanofillers, which automatically adjust the film permeability upon temperature elevation due to cold-chain interruption, retarding microbial growth; additionally, a temperature-triggered accelerated degradation mechanism ensures that the packaging rapidly enters the degradation pathway after failure [155,156]. The synergistic design of these intelligent functions and degradation kinetics drives starch-based packaging to evolve from passive protection toward active response and controllable degradation, providing a technical route for precisely matching the requirements of diverse food categories.

### 6.2. Other Potential Applications

Starch-based biodegradable packaging materials also demonstrate broad prospects in application fields beyond food packaging. In the pharmaceutical packaging sector, stringent requirements are imposed on biocompatibility and controllable degradation. Starch-based films have been explored for use in blister packaging and pharmaceutical capsule shells, where their non-toxicity and tunable degradation rates can match the release cycles of different drugs; nanomodification further imparts antimicrobial functionality, making them suitable for wound dressings and medical barrier films [155,156,157,158]. In the agricultural packaging sector, significant attention has been directed toward the application potential of starch-based seedling trays and mulch films. Their complete biodegradability avoids long-term soil pollution caused by conventional plastic residues, while precise regulation of degradation rates can match crop growth cycles; specifically, seedling trays require rapid degradation after use to facilitate transplanting, whereas mulch films must maintain structural integrity throughout the crop growth period and undergo rapid disintegration after harvest [159,160]. Additionally, the exploration of starch-based materials in areas such as slow-release fertilizer coatings and seed coatings likewise relies on their tunable degradation behavior and environmentally friendly characteristics.

## 7. Challenges and Future Perspectives

### 7.1. Current Key Challenges

Despite substantial progress, the cleaner-production transition of starch-based biodegradable packaging is constrained by several interrelated technical, environmental, and industrial barriers.

Precise control over degradation kinetics remains difficult to achieve, as existing modification strategies struggle to realize the temporal matching of material stability during service life with rapid degradation at end-of-life. The consistency of degradation behavior across different environmental scenarios such as soil, compost, and marine environments remains inadequate, failing to meet the diverse degradation rate requirements of various food packaging applications.

The compatibility and cost bottlenecks of nanomodification technologies have yet to be fundamentally resolved. Hydrophilic nanofillers tend to aggregate, while hydrophobic fillers require complex surface modification, both of which escalate production costs. Moreover, most studies remain confined to laboratory scales, lacking economically feasible large-scale preparation processes.

The environmental impact assessment framework remains incomplete. Existing life-cycle assessments lack unified methodological standards, and there is insufficient evaluation of nanoparticle ecotoxicity, the environmental fate of degradation products, and long-term risks associated with improper disposal scenarios such as landfilling and uncontrolled environmental release, hindering comprehensive life-cycle sustainability certification of the materials.

Industrial translation and cleaner production technologies are underdeveloped. Starch-based materials exhibit poor hydrothermal processing stability and substantial batch-to-batch performance variability, necessitating improved compatibility with existing plastic processing lines. Dedicated processing equipment and process specifications tailored to nanomodified systems remain lacking.

Performance gaps relative to conventional plastic packaging also persist, particularly in high-barrier properties such as oxygen and water vapor barrier under high-humidity conditions, heat seal strength, and long-term storage stability, making it difficult to fully substitute established petroleum-based packaging materials including polyethylene and polypropylene.

Addressing these challenges collectively is critical for advancing starch-based packaging materials from the laboratory to the market and realizing their genuine environmental benefits.

### 7.2. Future Research Trends

Looking toward the future development of starch-based biodegradable packaging materials, several frontier directions warrant in-depth exploration.

Precise regulation of smart responsive degradation kinetics requires the development of pH, enzyme, or thermosensitive nanoswitches to enable service stability followed by environmentally triggered rapid degradation, thereby decoupling material lifespan from disposal conditions and overcoming the inherent coupling between degradation behavior and environmental variability.

The synergistic development of green and cost-effective nanomodification involves advancing in situ nanofiller synthesis from agricultural waste, water-based solvent-free processing, and biomimetic surface modification to simultaneously reduce environmental footprint, enhance interfacial compatibility, and achieve balanced trade-offs among performance, cost, and sustainability.

Multifunctional integration in blend systems involves embedding antimicrobial, antioxidant, and freshness-indicating functions within starch-based matrices to create active and intelligent packaging composites, addressing the dual requirements of food safety and supply chain traceability in high-value applications.

Industrial adaptation of scalable modification processes requires establishing continuous extrusion compounding, online nanodispersion, and reactive blending technologies, supported by process–structure–degradation correlation models, to provide reliable processing windows for large-scale manufacturing.

Standardization of life cycle environmental impact assessment requires constructing a unified evaluation framework that incorporates nanoparticle ecotoxicity, degradation product fate, and multi-scenario disposal pathways to underpin green certification and steer material design toward verifiable life-cycle sustainability.

Elucidation of multiscale structure–degradation relationships involves combining in situ characterization, molecular dynamics simulations, and machine learning to uncover how nanomodification, blend architecture, and crystalline structure govern degradation kinetics, thereby enabling predictive design of degradation behavior.

The synergistic advancement of these research directions will propel starch-based packaging materials from mere biodegradability toward intelligently controlled degradability, from laboratory feasibility toward industrial reliability, ultimately realizing the sustainable development vision of substituting petroleum-based plastic packaging.

## 8. Conclusions

Starch-based packaging offers a promising route for reducing dependence on petroleum-derived plastics because it combines renewable feedstock availability, low cost, and inherent biodegradability. However, its cleaner-production potential can only be realized when hydrophilicity, insufficient mechanical strength, unstable barrier performance, and poorly controlled degradation are addressed together. This review shows that the multiscale structure of starch, from molecular chains and crystalline domains to granular morphology, governs enzymatic accessibility and degradation kinetics. Biological, physical, and chemical degradation pathways interact with starch structure, blend composition, nanofiller properties, and environmental conditions, making degradation control a central design requirement rather than only an end-of-life attribute. Nanomodification has evolved from single-filler reinforcement toward synergistic and green strategies that can improve matrix compatibility, barrier performance, mechanical stability, and degradation regulation. From a life-cycle perspective, starch-based packaging can reduce fossil-resource dependence and carbon burden, but agricultural inputs, processing energy, nanofiller release, degradation-product toxicity, and disposal conditions must be assessed consistently. Future research should therefore prioritize scalable green nanomodification, smart responsive degradation, multifunctional blend systems, low-carbon continuous processing, standardized life-cycle assessment, and predictive structure–property–degradation models. These advances are essential for converting starch-based packaging from a biodegradable material option into a cleaner-production platform with measurable environmental advantages and practical industrial relevance.

## Figures and Tables

**Table 1:** Major degradation pathways of starch-based packaging materials.

Pathway	Core Mechanism	Key Structural Changes	Final Products	Synergistic Effect	References
Biodegradation	Microbial extracellular enzymatic hydrolysis	Cleavage of α-1,4 and α-1,6 glycosidic bonds; gradual chain shortening	CO_2_, H_2_O, microbial biomass	Provides primary decomposition; accelerated by physical/chemical pretreatment	[58,59,60,61,62]
Physical degradation	Temperature, humidity, UV irradiation	Chain mobility increase; surface oxidation; structural loosening; chain scission	Lower molecular weight; hydrophilic surface	Improves enzyme accessibility; promotes chemical hydrolysis	[63,64,65,66]
Chemical degradation	Non-enzymatic hydrolysis and oxidation	Glycosidic bond cleavage; carbonyl/carboxyl formation; reduced crystallinity	Oxidized derivatives; fragmented chains	Increases hydrophilicity and disorder; boosts biodegradation	[67,68,69,70]

**Table 2:** Classification, mechanisms, and cleaner-production strategies of nanomodification technologies for starch-based materials.

Strategies	Type	Representative Nanomaterials	Key Mechanisms	Performance Outcomes	References
Nanofiller	One-dimensional (1D)	Cellulose nanofibers (CNF), Cellulose nanocrystals (CNC)	Formation of hydrogen bonding networks with starch matrix; restriction of molecular chain mobility; impediment of water permeation and enzyme diffusion	Enhanced interfacial adhesion; modulated degradation kinetics; improved mechanical properties	[99,100]
	Two-dimensional (2D)	Montmorillonite (MMT), MXene, Graphene oxide (GO)	Formation of physical barrier structures; prolongation of diffusion pathways for water vapor and gases	Improved barrier performance; reduced oxygen and water vapor transmission rates	[22,101,102]
	Zero-dimensional (0D)	Silver nanoparticles (AgNPs), Titanium dioxide (TiO_2_)	Antimicrobial activity (AgNPs); photocatalytic generation of reactive oxygen species (ROS) under UV (TiO_2_)	Inhibition of microbial proliferation; accelerated chain scission during end-of-life phase	[61,89,103]
Synergistic modification	Physical–nano synergy	Ultrasound-assisted treatment and nanofillers	Ultrasonic cavitation disrupts agglomerates; shear fields induce chain orientation; thermal profiles enhance interfacial wetting	Improved dispersion; anisotropic structures; reduced interfacial defects	[106,107,108]
	Chemical–nano synergy	Esterified/etherified starch and CNC; Crosslinking and layered silicates; Graft copolymerization	Hydrophobic moiety introduction; network anchoring; covalent attachment of polymer chains to filler surfaces	Dual reinforcement; improved interfacial adhesion; enhanced water vapor barrier	[109,110,111]
	Enzymatic–nano synergy	Amylase/transglycosidase and nanocellulose	Selective hydrolysis of amylopectin; rearrangement of chain length and branching structure	Enhanced filler efficiency; tunable degradation half-life	[112,113]
	Blending–nano synergy	Starch/PLA/CNC; Starch/MMT	Functional compartmentalization; preferential localization at interfacial regions	Delayed–accelerated–completed three-stage degradation; medium-to-long shelf-life suitability	[101,114]
Green nanomodification	Agricultural waste-derived	CNC/CNF from rice husks, wheat straw, corn stover, bagasse, banana pseudostems, and pineapple leaves	Waste-to-resource circulation; acid hydrolysis or mechanical extraction	Reduced feedstock costs; comparable reinforcement efficiency	[115,116,117]
	Green solvent-assisted	Ionic liquids; Aqueous systems (high-pressure homogenization); Supercritical CO_2_	Simultaneous starch dissolution and filler dispersion; solvent recovery; organic solvent-free processing	Homogeneous modification; scalable preparation; simplified process flow	[118,119,120]
	Biobased nanomodifiers	Chitosan nanoparticles; Starch nanocrystals; Lignin nanoparticles	Ionic gelation (chitosan); acid/enzymatic hydrolysis (starch); intrinsic antimicrobial and antioxidant activity	Complete biodegradability; excellent interfacial compatibility; no secondary pollution	[121,122,123]

## Data Availability

No new data were created or analyzed in this study. Data sharing is not applicable to this article.

## Abbreviations

The following abbreviations are used in this manuscript:

0D	Zero-dimensional
1D	One-dimensional
2D	Two-dimensional
AgNPs	Silver nanoparticles
CNC	Cellulose nanocrystal
CNF	Cellulose nanofiber
GO	Graphene oxide
MMT	Montmorillonite
PE	Polyethylene
PET	Polyethylene terephthalate
PLA	Polylactic acid
PP	Polypropylene
PVA	Polyvinyl alcohol
ROS	Reactive oxygen species
TiO_2_	Titanium dioxide
UV	Ultraviolet
